# Amino acid substitutions at the G118 hotspot in dihydroorotate dehydrogenase confer olorofim resistance in *Aspergillus flavus*

**DOI:** 10.1128/spectrum.00251-26

**Published:** 2026-07-14

**Authors:** Hui Xu, Tianyu Liang, Zhongwei Wang, Yalu Wei, Zhe Wan, Ruoyu Li, Liangren Zhang, Wei Liu

**Affiliations:** 1 Department of Dermatology and Venerology, Peking University First Hospital 26447, Beijing, China; 2 National Clinical Research Center for Skin and Immune Diseases 71044 , Beijing, China; 3 Research Center for Medical Mycology, Peking University 12465, Beijing, China; 4 Beijing Key Laboratory of Molecular Diagnosis on Dermatoses 12465, Beijing, China; 5 State Key Laboratory of Natural and Biomimetic Drugs, School of Pharmaceutical Sciences, Peking University 26447, Beijing, China; Universidade de Sao Paulo Campus de Ribeirao Preto, Ribeirao Preto, Sao Paulo, Brazil

**Keywords:** *Aspergillus flavus*, olorofim, resistance, CRISPR-Cas9, molecular docking

## Abstract

The clinical management of invasive aspergillosis (IA) is limited by the scarcity of antifungal agents and the emergence and spread of drug resistance, which impairs therapeutic efficacy and poses a significant public health threat. Olorofim, a first-in-class clinical antifungal targeting dihydroorotate dehydrogenase (DHODH), represents a promising therapeutic option for IA. It shows activity against several filamentous fungi, including *Aspergillus* spp., both *in vitro* and *in vivo*. Olorofim-resistant *Aspergillus fumigatus* isolates, characterized by various amino acid substitutions (with a hotspot at G119), have been generated under laboratory conditions through olorofim exposure. However, the potential olorofim resistance mechanisms in *Aspergillus flavus*, the second-most common pathogen causing IA globally, remain entirely unexplored. In this study, we revealed that the residue G118 in *A. flavus*, conserved in many filamentous fungi, is homologous to G119 in *A. fumigatus* by DHODH amino acid sequence alignment. Using a CRISPR-based point mutation approach, we further confirmed that substitutions at the DHODH G118 residue confer acquired olorofim resistance in *A. flavus*. Additionally, we established a *Galleria mellonella* infection model and observed that these resistant isolates exhibited no obvious fitness cost, and olorofim treatment was ineffective against infections caused by DHODH G118 mutants. Molecular docking models further indicated that substitutions at the G118 locus may introduce steric hindrance, reducing the binding affinity between olorofim and the DHODH active site and thereby driving resistance.

**IMPORTANCE:**

Invasive aspergillosis, a life-threatening infection in immunocompromised patients, is increasingly difficult to treat due to limited antifungal options and rising resistance. Olorofim, a novel antifungal targeting dihydroorotate dehydrogenase (DHODH), represents a promising therapy. While resistance in *Aspergillus fumigatus* has been linked to DHODH G119 mutations, the mechanisms in the globally prevalent pathogen *Aspergillus flavus* remained unknown. Using CRISPR-based mutagenesis, we demonstrate that amino acid substitutions at G118 confer olorofim resistance, supported by molecular docking showing reduced drug binding. Importantly, olorofim failed to protect larvae infected with resistant mutants in an infection model. These findings provide essential insights for proactively monitoring and managing olorofim resistance in *Aspergillus flavus*, aiding clinical and public health responses.

This study is registered with ClinicalTrials.gov as NCT05101187 and NCT06969703.

## INTRODUCTION

Invasive aspergillosis (IA) is the most severe form of *Aspergillus* infection, primarily affecting critically ill patients, individuals with chronic pulmonary diseases, and immunocompromised populations, such as those with malignancies or organ transplants (1). While *Aspergillus fumigatus* is the most common cause of IA globally, infection due to *Aspergillus flavus* is predominant in Asia, the Middle East, and Africa, potentially owing to the better ability of this species to survive in hot and arid climatic conditions compared to other *Aspergillus* species (2). In addition to its role as a pathogenic fungus, *A. flavus* is a major concern due to its production of aflatoxins, which pose significant risks to food safety and human health.

Currently, treatment options for IA are limited. For primary therapy, voriconazole (VRC) and isavuconazole (ISA) are recommended as the mainstay agents, with liposomal amphotericin B (AMB) as an alternative; posaconazole (POS) is mainly used for prophylaxis, while echinocandins and other agents are generally reserved for salvage therapy or selected combination regimens in refractory disease (3). Notably, *A. flavus* possesses intrinsic resistance to certain antifungals, such as fluconazole and flucytosine. Furthermore, compared to *A. fumigatus*, it exhibits distinct resistance patterns, most prominently a reduced susceptibility to AMB (4). However, with the widespread global use of triazole antifungals and agricultural fungicides, the prevalence of triazole-resistant *Aspergillus* strains has increased, significantly complicating IA management (5–7). Furthermore, current antifungal therapies are limited by drug delivery constraints (8), adverse effects (9), and drug-drug interactions (10). These challenges, coupled with the escalating issue of antifungal resistance, underscore the urgent need for novel antifungal agents to improve IA management (11).

Olorofim (F901318) is an innovative antifungal drug to be developed in nearly 20 years, featuring a unique mechanism of action distinct from existing antifungals (12). It selectively inhibits fungal dihydroorotate dehydrogenase (DHODH), a key enzyme in pyrimidine biosynthesis, leading to the depletion of essential substrates for cellular processes, including cell wall integrity and DNA replication (12). *In vitro* susceptibility testing has shown that olorofim is highly active against all pathogenic *Aspergillus* spp. (13), *Scedosporium* spp., *Lomentospora prolificans* (14), and dimorphic fungi (e.g., *Coccidioides* spp. [14] and *Talaromyces marneffei* [15]). It has been granted orphan drug designation and breakthrough therapy designation by the European Medicines Agency and the U.S. Food and Drug Administration for treating various invasive fungal diseases (IFDs) (16). Recently, a phase 2b clinical study demonstrated that olorofim is effective and well tolerated in patients with IFDs who lack alternative treatment options (17). Although olorofim is currently undergoing a phase 3 trial (NCT05101187), it has already been used to treat patients with refractory filamentous fungal infections successfully in clinical practice (18–20).

However, olorofim-resistant *A. fumigatus* isolates have been obtained under laboratory conditions using high concentrations of olorofim. Sequencing of the DHODH-encoding gene *pyrE* has revealed that DHODH’s G119 locus is a hotspot for olorofim resistance in *A. fumigatus* (21), with G119C, G119S, and G119V representing the most frequent amino acid substitutions at this locus. Additionally, ipflufenoquin, a novel agricultural fungicide that targets the same enzyme as olorofim, has been approved by the U.S Environmental Protection Agency and the Australian Pesticides and Veterinary Medicines Authority. Strains resistant to ipflufenoquin, selected after exposure to ipflufenoquin, also exhibit resistance to olorofim (22). This resistance mechanism involves point mutations at multiple sites in the DHODH protein, including the G119 residue. Similar to the cross-resistance to medical triazoles driven by environmental exposure to azole fungicides, the cross-resistance between ipflufenoquin and olorofim suggests that widespread agricultural use of ipflufenoquin may compromise the clinical efficacy of olorofim.

However, existing studies on olorofim resistance have primarily focused on *A. fumigatus*, and the underlying mechanisms in *A. flavus* remain entirely unknown. In this study, we first generated *A. flavus* strains harboring DHODH G118 substitutions via CRISPR-Cas9-mediated genome editing and characterized the phenotypic traits of these mutants, including antifungal susceptibility profiles, growth rates, and adaptability under various stress conditions. Subsequently, the pathogenicity of these mutants and the *in vivo* therapeutic efficacy of olorofim were evaluated using a *Galleria mellonella* infection model. Finally, molecular docking analyses of olorofim bound to *A. flavus* DHODH were performed to elucidate the underlying resistance mechanism.

## MATERIALS AND METHODS

### Amino acid sequence alignment

The DHODH protein sequences of *A. fumigatus* (Q4X169_ASPFU), *A. flavus* (B8NFW7_ASPFN), *L. prolificans* (A0A2N3N4H6_9PEZI), *Coccidioides posadasii* (C5PJ23_COCP7), and *T. marneffei* (B6QRM1_TALMQ) were obtained from the UniProt Knowledgebase (https://www.uniprot.org/) and aligned using CLUSTALW (https://www.genome.jp/tools-bin/clustalw). Conserved residues were highlighted in black, and the G118 residue of *A. flavus* DHODH (*Afl*DHODH) and the corresponding residues in the other species were labeled with red triangles.

### Construction of *A. flavus* DHODH G118 substituted strains

#### crRNA design, RNA duplex, and Cas9 nuclease

To evaluate the impact of DHODH G118 substitution on susceptibility to olorofim, we introduced G118C, G118S, and G118V mutations into *A. flavus* NRRL3357 using a marker-free CRISPR-Cas9 method (23). Using EuPaGDT (http://grna.ctegd.uga.edu/), a protospacer adjacent motif near the G118 and a 20 bp protospacer covering the locus were selected based on the *pyrE* gene (NCBI gene ID: 7911021); the highest-scoring crRNA was chosen (Table 1). Next, 100 bp single-stranded DNA repair templates were synthesized centered on the target, with codon GGG replaced by TGT (C), AGC (S), or GTG (V). The CRISPR-Cas9 targeting and repair strategy is illustrated in Fig. S1a. TracrRNA, crRNA, and Cas9 nuclease were ordered from Integrated DNA Technologies (IDT, Inc., Coralville, IA, USA).

**TABLE 1:** crRNA, repair templates, and primers used in this study^*a*^

Name	Sequence (5′→3′)	Purpose
crRNA	CGGAGGATGCGCACCATATT	CRISPR-Cas9 guide RNA
Repair template (G118C)	CCTCTGATCCGGCTGTTGTACCCTGATGCGGAGGATGCGCACCATATTTGTGTGGATGCTTTGAAGAATCTTTACAAGCTTGGACTCCACCCCAGA	Repair template
Repair template (G118S)	CCTCTGATCCGGCTGTTGTACCCTGATGCGGAGGATGCGCACCATATTAGCGTGGATGCTTTGAAGAATCTTTACAAGCTTGGACTCCACCCCAGA	Repair template
Repair template (G118V)	CCTCTGATCCGGCTGTTGTACCCTGATGCGGAGGATGCGCACCATATTGTGGTGGATGCTTTGAAGAATCTTTACAAGCTTGGACTCCACCCCAGA	Repair template
PyrE_G118_Fwd	TTCATGTGGTACGATGCTCT	PCR amplification and sequencing for *pyrE*
PyrE_G118_Rev	TCTTTTTTTTTCTCTCTTTGCTTC	PCR amplification and sequencing for *pyrE*
Asp1S	ATGCCTGTCCGAGCGT	TaqMan real-time PCR
AflR2	TTAAGTTCAGCGGGTATRCC	TaqMan real-time PCR
AflP	FAM-CGCTTGCCGAACGCAAATCAATCTT-TAMRA	TaqMan probe

^*a*^ In primer AflR2, R is a degenerate base representing A or G.

#### CRISPR-Cas9-mediated point mutation in *pyrE* gene

*A. flavus* protoplasts were transformed following a published protocol (24). Briefly, fungal spores (1 × 10⁶) were cultured in 100 mL of Sabouraud dextrose broth (SDB) at 37°C for 4–6 h. Transformation solutions included TRAFO 1 (0.6 M KCl, 50 mM CaCl_2_, 5 mM Tris-HCl, pH 7.5), TRAFO 2 (40% PEG3350 in TRAFO 1), and filter-sterilized Cas9 buffer (20 mM HEPES, 150 mM KCl, pH 7.5). After incubation, spores were harvested by centrifugation (3,000 × *g*, 5 min), washed with SDB, and resuspended in 40 mL of digestion solution containing 2.8 g NaCl, 100 mg Driselase, 400 mg lysing enzymes, 0.8 mL β-glucuronidase, 20 mM CaCl_2_, and 20 mM phosphate-buffered saline. Protoplasts were generated by horizontal shaking (30°C, 70 rpm, 4 h). The resulting mixture was then centrifuged (3,000 × *g*, 5 min, RT) to remove the supernatant. The protoplasts were sequentially resuspended in 10 mL and then 1 mL of TRAFO 1, with centrifugation repeated after each step. Protoplasts were counted and adjusted to 5 × 10⁶/mL. The Cas9 ribonucleoprotein complex was assembled by combining 0.17 μL of 33 μM gRNA, 0.17 μL of 1 μg/μL Cas9 nuclease, and 2.5 μL Cas9 working buffer, followed by 5 min pre-incubation. This complex was mixed with 105 μL protoplasts, 0.5 μg–1 μg repair template DNA, and 25 μL TRAFO 2, then incubated on ice for 50 min. After adding 250 μL TRAFO 2 and incubating for 20 min at RT, transformations were resuspended in olorofim-containing top agar (10.56 g [NH₄]₂SO₄, 1.4 g agarose, 68.4 g sucrose, 9.2 g SDB, adjusted to 200 mL with distilled water, autoclaved), and plated onto hypertonic agar substrate (13.2 g [NH₄]₂SO₄, 85 g sucrose, 24 g SDA, 400 mL distilled water, autoclaved) supplemented with 1 µg/mL olorofim (MedChemExpress Co. Ltd., CAS no.: 1928707-56-5). Protoplasts without repair templates were plated onto olorofim-containing or standard hypertonic agar for negative/positive controls.

Conidia were harvested after 3 days and pooled from three to four plates. Transformants (10³–10⁵ CFU) were plated on potato dextrose agar (PDA) containing 0.25 µg/mL–2 µg/mL olorofim; nontransformed controls were plated on drug-free PDA. After 3 days at 37°C, four resistant and one sensitive colonies were streaked onto fresh PDA. Single colonies were isolated after 2 days of growth, and conidia were expanded on new PDA plates to obtain purified strains for sequencing and phenotypic validation.

#### Genomic DNA extraction and verification of the *A. flavus pyrE* gene by sequencing

Genomic DNA was extracted with the Biospin Fungus Genomic DNA Extraction Kit (Bioer Technology Co., Ltd., Hubei, China). The *A. flavus pyrE* gene was amplified using primers PyrE_G118_Fwd and PyrE_G118_Rev (Table 1). The PCR products were validated for size by agarose gel electrophoresis and sequenced by BGI Genomics. Sanger sequencing results for the strains harboring point mutations are shown in Fig. S1b.

### *In vitro* antifungal susceptibility testing

Antifungal susceptibility testing was performed by the broth microdilution method following CLSI M38-A3 guidelines (25). The tested drugs included ITC, VRC, POS, ISA, caspofungin (CAS), AMB (all from Harvey Biotech Co., Ltd., Beijing, China), ipflufenoquin (MedChemExpress, USA), and olorofim. Final concentration ranges were 0.0313 µg/mL–16 µg/mL for ITC, VRC, POS, ISA, AMB, and olorofim; 0.008 µg/mL–4 µg/mL for CAS; and 0.06 µg/mL–32 µg/mL for ipflufenoquin. *A. flavus* (ATCC 204304) was used as the quality control strain. All isolates were subcultured on PDA at 35°C for 3–4 days to induce conidium formation. Conidial suspensions were prepared in sterile 0.1% (vol/vol) Tween-20, allowed to settle for 3–5 min. The upper homogeneous suspensions were taken and diluted in RPMI 1640 medium to 10^4^ CFU/mL. Inoculated 96-well plates were incubated at 35°C, with minimum effective concentrations (MECs) determined at 24 h and minimum inhibitory concentrations (MICs) at 48 h (26). For CAS, the MEC was defined as the lowest concentration that induced minimal, circular, compact hyphal growth compared with the growth control. For triazoles, AMB, and olorofim, the MIC was the lowest concentration that resulted in complete inhibition of fungal growth relative to the growth controls.

Disk diffusion testing was conducted as previously described (27). In-house prepared disks loaded with 30 µg olorofim were placed in the center of the inoculated agar plates. The plates were incubated at 35°C for 48 h, after which the resulting inhibition zone diameters were measured.

### *In vitro* fitness cost assays

#### Radial growth rate testing

A standardized inoculum of 2 × 10² spores was inoculated centrally on PDA plates. Each strain was tested in triplicate, and mean colony diameters were measured along two perpendicular axes every 24 h for 6 days at 35°C. Day 6 growth rates (mm/day) were calculated, and statistical significance was evaluated using Student’s *t*-test (*P* < 0.05).

#### Spot assays

For stress resistance assays, autoclaved PDA was cooled to approximately 50°C before adding stress-inducing agents alone or with olorofim. Stress-inducing agents included Congo red (600 μg/mL), calcofluor white (300 μg/mL), SDS (0.03%), H_2_O_2_ (3 mM), and menadione (80 mM). For combinatorial assays, these agents were added to PDA medium supplemented with olorofim at two concentrations (0.005 μg/mL and 4 μg/mL). Serially diluted *A. flavus* spores (2 × 10⁵ to 2 spores) were inoculated onto plates. Plates were incubated at 35°C for 48 h to assess growth inhibition.

### *In vivo* assessment of *A. flavus* infection in a *G. mellonella* model

#### Model establishment and therapeutic intervention

*G. mellonella* has been validated as a reliable preliminary model for assessing the pathogenicity and antifungal susceptibility of *Aspergillus* spp., with results highly concordant with mammalian models (28). The *G. mellonella* infection model was established as previously described (29), with modifications. Briefly, each larva was injected with 20 μL of conidial suspension (5 × 10⁵ CFU/mL in PBS) into the left posterior proleg. Survival was monitored at 12 h intervals for 7 days post-infection. For therapeutic assessment, larvae were grouped by infection with NRRL3357 or DHODH-substituted mutants. Olorofim treatment (8 nmol/dose) started at 4 h post-inoculation, with three administrations at 4, 28, and 52 h (30). All experiments included three biological replicates (*n* = 30/group). Statistical significance was determined via log-rank (Kaplan-Meier) test in GraphPad Prism 10 (*P* < 0.05).

#### Quantitative real-time PCR for fungal burden assessment

Serially diluted *A. flavus* spores (1 × 10⁸ to 1 × 10³ CFU) were added to uninfected *G. mellonella* larvae (*n* = 5/group). DNA was extracted using DNeasy Blood & Tissue Kit (QIAGEN, Hilden, Germany) and quantified by NanoDrop 2000. TaqMan real-time PCR targeting the *A. flavus* internal transcribed spacer region was performed using the forward primer Asp1S, reverse primer AflR2, and probe AflP (Table 1). The primers and probe were previously validated for high sensitivity and specificity (31). PCR was performed on an Applied Biosystems ViiA 7, and Ct values were plotted against log_10_ CFU per larva to generate a standard curve (Ct = −0.3384 × log_10_ CFU per larva + 11.317, *R*² = 0.9608). For fungal burden assessment, DNA was extracted from five larvae/group, and Ct values were converted to CFU using the standard curve. Group differences were analyzed using two-way ANOVA (*P* < 0.05 was considered significant).

#### Histopathological analysis

Histopathological examination was performed to assess the *in vivo* efficacy of olorofim in *G. mellonella* larvae infected with mutant strains. Larvae infected with mutant strains (with or without olorofim treatment) were compared with the PBS-injected control group. Three larvae per group were collected at 3 days post-infection, fixed in 4% paraformaldehyde for 72 h with agitation, and embedded in paraffin for hematoxylin and eosin (H&E) staining. Fungal burden, distribution, and hyphal morphology were observed by bright-field microscopy.

### *In silico* protein prediction, docking, and molecular dynamics simulation

The human DHODH protein structure was obtained from the Protein Data Bank (PDB ID: 6QU7). The initial protein model of *Afl*DHODH was derived from the AlphaFold database on the UniProt website (UniProt ID: B8NFW7). Ligands (olorofim, orotate, and FMN) were saved as “.sdf” files by ChemDraw 22.2.0 and prepared by Schrödinger Suite 2018 with the “LigPrep” module. The protein model was prepared using the "Protein Preparation Wizard" module in Schrödinger Suite 2018, which involved a series of preprocessing steps, including hydrogenation, charge assignment, and structural refinement. The two generated protein models were aligned using the “Protein Structure Alignment” module in Schrödinger Suite 2018.

The “Receptor Grid Generation” module in Schrödinger Suite 2018 was used to define the ligand binding site in human DHODH (PDB ID: 6QU7) as the docking pocket, with fixed X, Y, and Z coordinates and a grid box size of 20 Å. These coordinates were then applied to the *Afl*DHODH protein for the next docking step. Ligands were docked into *Afl*DHODH via induced-fit docking using the corresponding module. Mutants were generated and interatomic distances were measured using PyMOL 2.5. Figures were prepared with Schrödinger Suite and PyMOL.

A 400 ns molecular dynamics (MD) simulation of the olorofim-protein complex was conducted in Desmond under NPT conditions (300 K, 1.01325 bar). To maintain electrostatic neutrality, Na^+^ and Cl⁻ ions were added at a physiological concentration of 0.15 M. The simulation was performed using the OPLS3 force field in combination with the SPC explicit solvent model, and system dynamics were recorded at 1.2 ps intervals. The model systems were equilibrated using Desmond’s six-step default relaxation protocol to ensure production-quality results. All other parameters were maintained at default settings for consistency. The Simulation Interaction Diagram analyzed energy changes, root-mean-square deviation fluctuations, hydrogen bonds, and van der Waals interactions throughout the trajectory.

## RESULTS

### The DHODH G118 locus of *A. flavus* is conserved in filamentous fungi

**Fig 1:**
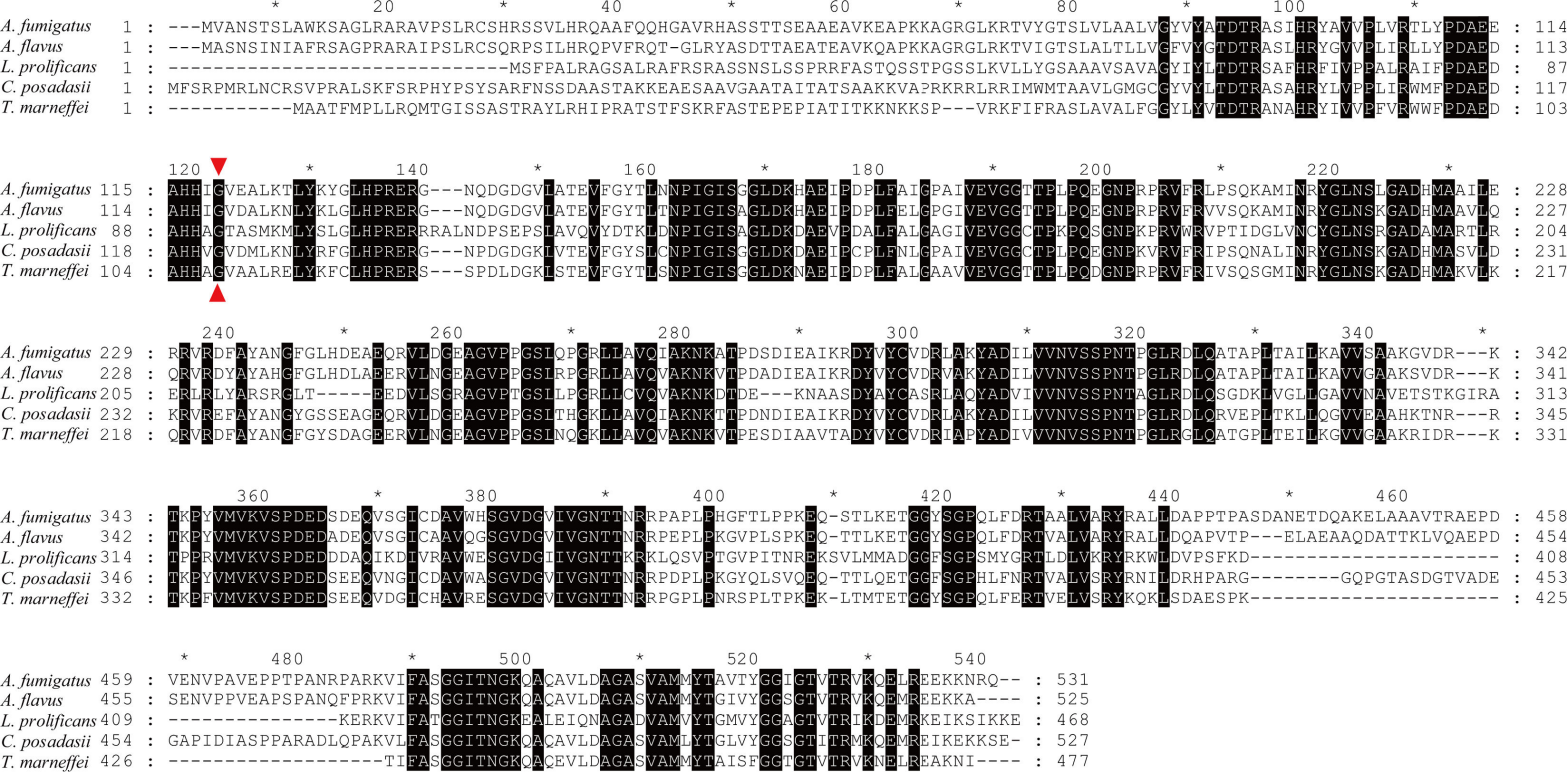
DHODH amino acid sequence alignment of *A. fumigatus, A. flavus, L. prolificans, C. posadasii,* and *T. marneffei*. The DHODH protein sequences were aligned using CLUSTALW. Conserved residues were highlighted in black, and the G118 residue of *Afl*DHODH and the corresponding residues in other species were labeled with red triangles. Asterisks denote position markers at 10-residue intervals.

We aligned the DHODH sequences of *A. fumigatus* and *A. flavus* and found that *A. fumigatus* DHODH (*Af*DHODH) consists of 531 amino acids, whereas *Afl*DHODH consists of 525 amino acids. The sequence identity between the two proteins was 80.15%. Notably, the G119 locus in *Af*DHODH corresponds to the G118 locus in *Afl*DHODH, suggesting that G118 may represent a mutation hotspot for olorofim resistance in *A. flavus*. To further investigate conservation, we aligned DHODH sequences from *A. fumigatus*, *A. flavus*, *L. prolificans*, *C. posadasii*, and *T. marneffei*, all of which are susceptible to olorofim and are pathogens against which olorofim is effective in treating IFDs. The analysis confirmed that the G118 locus of *Afl*DHODH is conserved across these species (Fig. 1), and this conservation implies that mutations at this locus may potentially confer olorofim resistance across these fungal species.

### DHODH G118 locus amino acid substitutions confer high-level olorofim resistance in *A. flavus*

**Fig 2:**
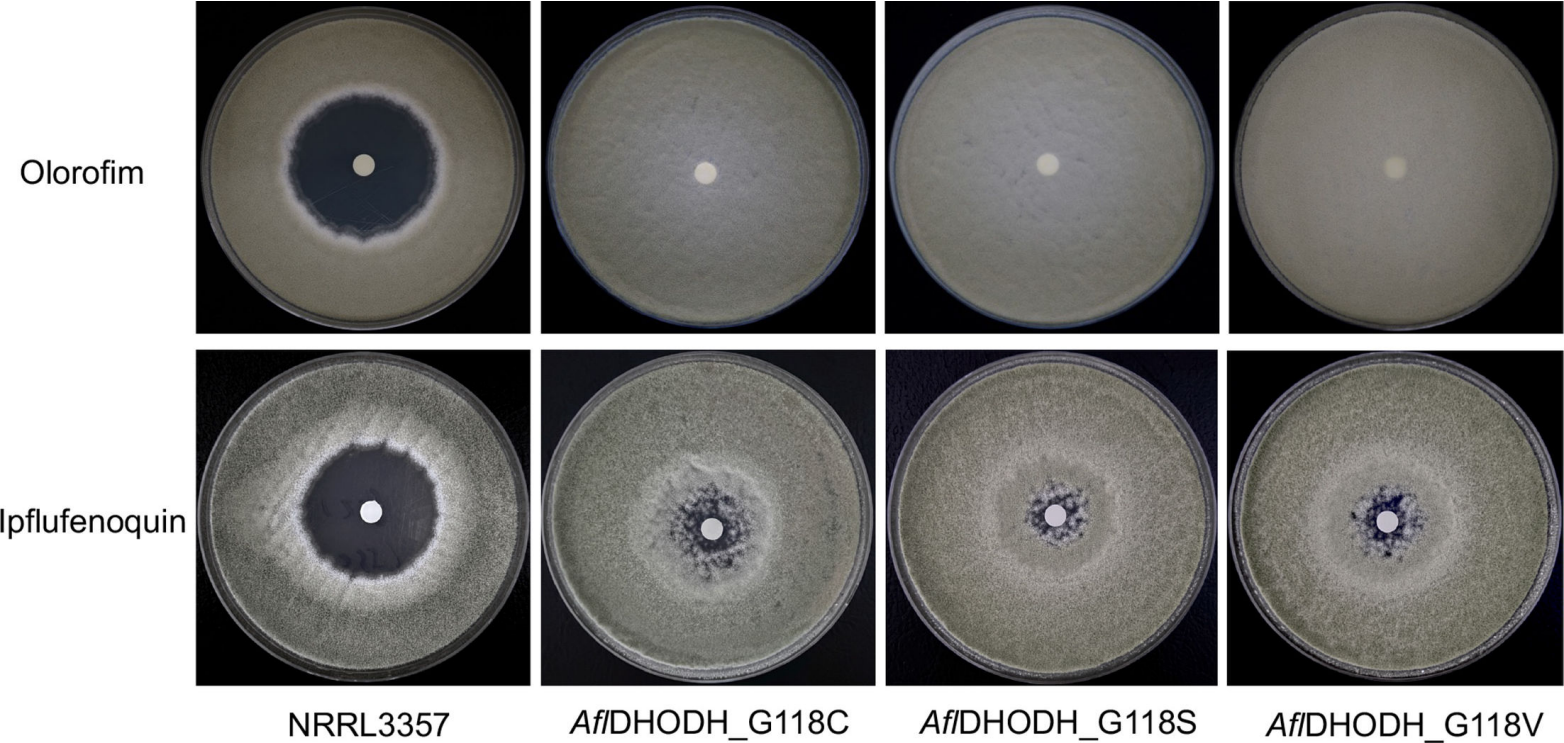
Antifungal susceptibility of *Afl*DHODH G118-substituted strains to olorofim and ipflufenoquin determined by disk diffusion assay. Inoculum suspensions were spread on 90 mm plates, and in-house disks containing 30 µg of olorofim and ipflufenoquin were placed at the center. Plates were incubated at 35°C for 48 h, and the inhibition zone diameters were measured.

To determine whether G118 substitutions in *Afl*DHODH confer resistance to olorofim, similar to the G119 substitutions in *Af*DHODH, we constructed mutant *A. flavus* strains by CRISPR-Cas9 method. Three mutant strains, *Afl*DHODH_G118C, *Afl*DHODH_G118S, and *Afl*DHODH_G118V, were obtained, in which G118 in DHODH was substituted with cysteine, serine, or valine, respectively. The antifungal susceptibility of these strains, along with the wild-type (WT) NRRL3357, was assessed using the broth microdilution method. Results showed that the MIC of olorofim for NRRL3357 was 0.03 µg/mL, whereas all G118-substituted strains exhibited MICs >16 µg/mL (Table 2). Similarly, the MIC of ipflufenoquin (DHODH-targeting fungicide) against NRRL3357 was 4 µg/mL, while all G118-substituted strains showed MICs >16 µg/mL. Notably, all mutant strains showed unchanged susceptibility to ITC, VRC, POS, ISA, AMB, and CAS. Disk diffusion assays for olorofim and ipflufenoquin showed significantly smaller inhibitory zones for G118 mutant strains compared with NRRL3357 (Fig. 2). These findings collectively demonstrate that G118 substitutions in *Afl*DHODH confer resistance to both olorofim and ipflufenoquin without altering sensitivity to other antifungals.

**TABLE 2:** *In vitro* antifungal susceptibility of *A. flavus* strains^*a*^

Strains	MIC/MEC (μg/mL)								Inhibition diameter (mm)	
	OLO	Ipflufenoquin	VRC	ITC	POS	ISA	AMB	CAS	OLO	Ipflufenoquin
NRRL 3357	0.03	4	0.5	0.12	0.12	0.5	1	0.03	22	16
*Afl*DHODH_G118C	>16	>16	0.5	0.12	0.12	0.5	1	0.03	0	0
*Afl*DHODH_G118S	>16	>16	0.5	0.12	0.12	0.5	1	0.03	0	0
*Afl*DHODH_G118V	>16	>16	0.5	0.12	0.12	0.5	1	0.03	0	0

^*a*^ OLO, olorofim; ITC, itraconazole; VRC, voriconazole; POS, posaconazole; ISA, isavuconazole; AMB, amphotericin B; CAS, caspofungin.

### Olorofim-resistant *A. flavus* harboring DHODH G118 substitutions causes olorofim treatment failure in the *G. mellonella* infection model

**Fig 3:**
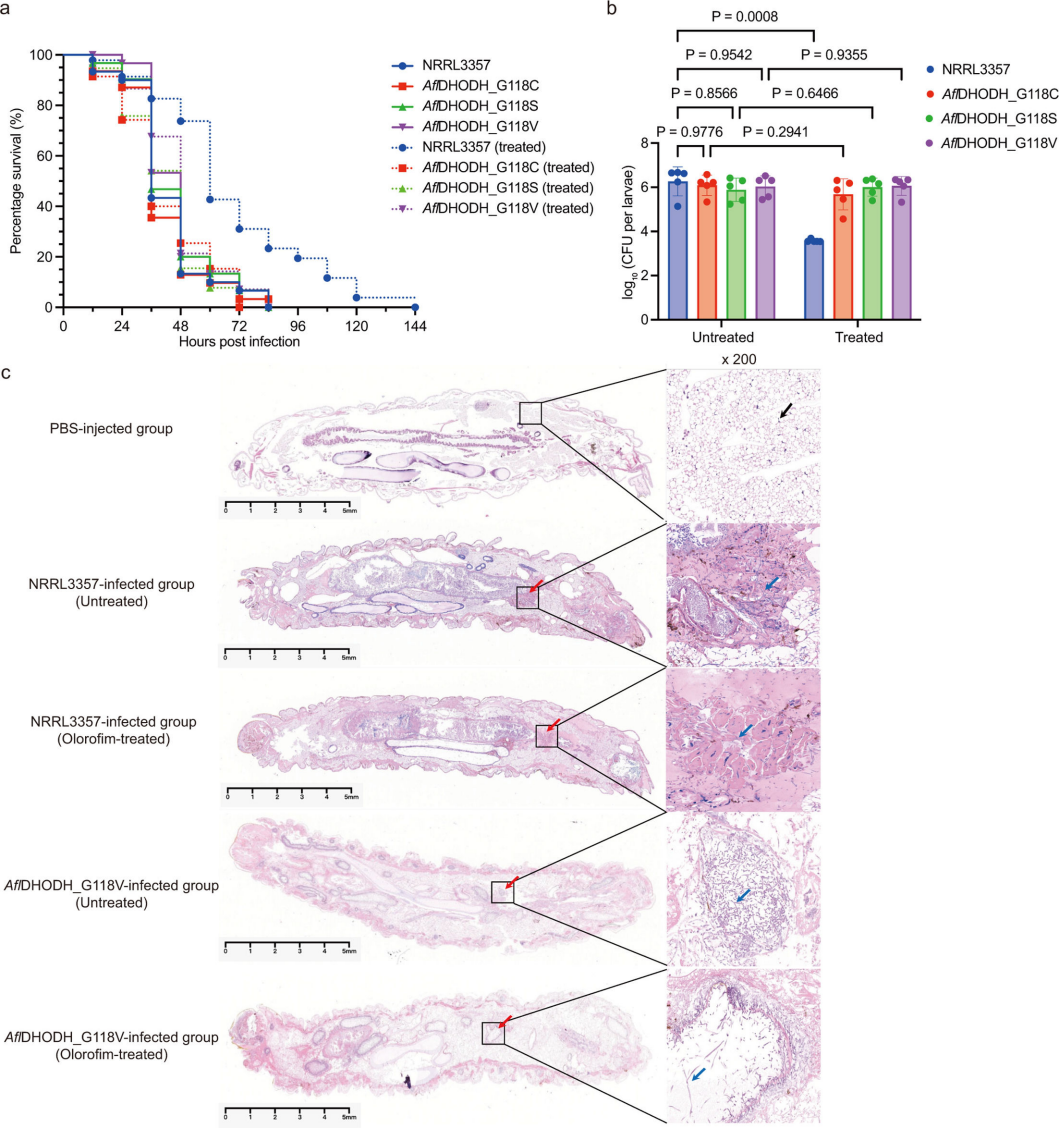
(**a**) Survival curves of *G. mellonella* larvae infected with NRRL3357, *Afl*DHODH_G118C, *Afl*DHODH_G118S, and *Afl*DHODH_G118V. Groups not receiving olorofim treatment are represented by solid lines, and those receiving olorofim treatment by dashed lines. Statistical differences between groups were analyzed using the log-rank test. (**b**) Fungal burden of larvae in different groups. DNA was extracted from five larvae per group, and the fungal burden was quantified using TaqMan probe-based real-time PCR. Ct values were converted to fungal load using a standard curve. Differences between groups were analyzed using two-way ANOVA. (**c**) Histopathological analysis of larval tissues. Black arrows indicate intact circular vacuoles; red arrows indicate disseminated hyphal nodules; blue arrows indicate acute-angle branching (<45°) of *A. flavus* hyphae.

To evaluate the impact of DHODH G118 substitutions on *in vivo* treatment efficacy, we assessed survival rates and fungal burdens using a *G. mellonella* infection model. Both WT and G118-substituted strains exhibited high pathogenicity, with all larvae dying within 4 days post-infection. There were no significant differences in survival curves among the untreated groups (*P* = 0.7720; Fig. 3a) or in fungal burden (*P* > 0.05; Fig. 3b). Following olorofim treatment, the median survival time of larvae infected with the WT strain significantly extended from 36 to 60 h (a 67% increase, *P* < 0.0001; Fig. 3a), with a significant reduction in fungal burden (*P* = 0.0008; Fig. 3b). In contrast, olorofim treatment failed to provide therapeutic benefits to larvae infected with G118-substituted strains, showing no significant extension in median survival time (Fig. 3a) or reduction in fungal burden (*P* > 0.05; Fig. 3b).

Histopathological analysis further confirmed these findings. In larvae infected with the WT strain, the untreated group exhibited dense hyphal masses. However, following olorofim treatment, the fungal burden was dramatically reduced, and the integrity of the host tissue architecture was largely preserved. In striking contrast, *G. mellonella* infected with the mutants revealed disseminated hyphal nodules across the entire field of view, with no observable reduction in fungal density regardless of olorofim treatment (Fig. 3c). Within these nodules, the hyphae exhibited classic *A. flavus* morphological features, including acute-angle branching (<45°), radially arranged septate hyphae, and antler-like terminal extensions. In contrast, larvae in the PBS control group displayed intact circular vacuoles, artifacts of lipid dissolution during H&E processing. These results suggested that *A. flavus* strains carrying G118 substitutions retain pathogenicity and cause treatment failure in the *G. mellonella* infection model. To determine whether the G118 substitutions incurred any *in vitro* fitness costs, we compared the growth rates of the WT and mutant strains (Fig. S2) and further tested their tolerance to various stressors, including cell wall-perturbing agents (Congo red, calcofluor white, and SDS) and oxidative stress inducers (H_2_O_2_ and menadione) (Fig. S3). No significant differences in growth or stress tolerance were observed between the mutants and the WT strain. These results indicate that the G118 mutations do not compromise the general fitness of *A. flavus*.

### *In silico* modeling suggests DHODH G118 substitutions may compromise the binding affinity of olorofim by introducing steric clashes

**Fig 4:**
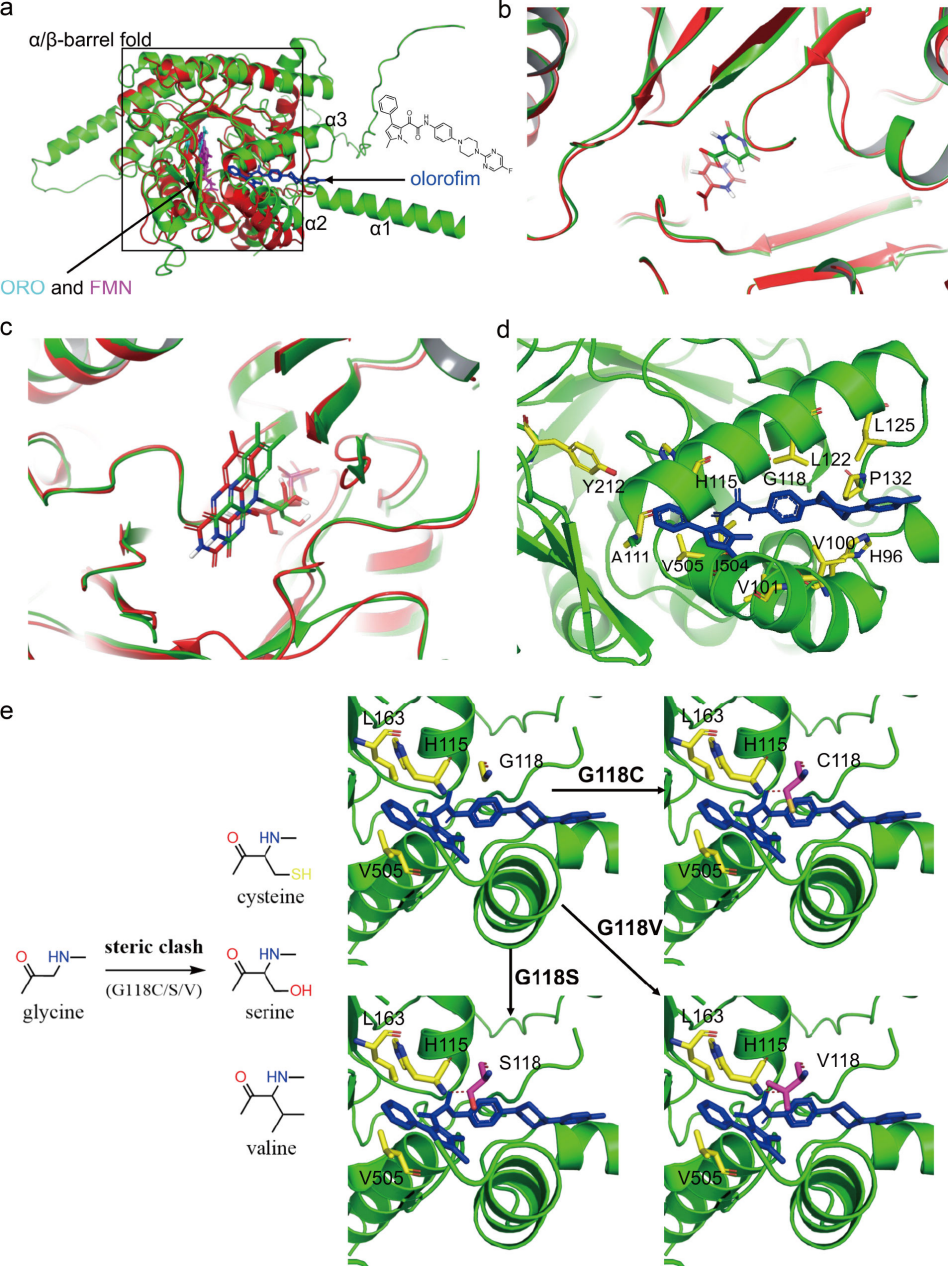
(**a**) Structural alignment of hDHODH (red) and *Afl*DHODH (green). Binding modes of ORO (cyan), FMN (magenta), and olorofim (blue) with *Afl*DHODH are shown. (**b**) The co-crystal structure of hDHODH (red) in complex with orotate (red) is presented, with the predicted binding pose of orotate (green) from molecular docking with *Afl*DHODH (green) aligned for comparison. (**c**) The co-crystal structure of hDHODH (red) in complex with FMN (red) is presented, with the predicted binding pose of FMN (green) from molecular docking with *Afl*DHODH (green) aligned for comparison. (**d**) 3D diagram illustrating receptor-ligand interactions between olorofim and *Afl*DHODH. (**e**) Substitutions of G118 in *Afl*DHODH (green) with C118, S118, or V118 increase steric hindrance between olorofim (blue) and *Afl*DHODH. Olorofim and key residues (yellow and pink) are shown as sticks. Steric clashes are represented by red dashed lines. Mutant models were generated using PyMOL.

Lacking a crystal structure for *Afl*DHODH, we performed comparative structural analysis by aligning the AlphaFold-predicted *Afl*DHODH model (AF-B8NFW7-F1) with the human DHODH (hDHODH) crystal structure (PDB: 6QU7). Using Schrödinger Suite 2018, we aligned protein backbones and obtained an alignment score of 0.136 and a root mean square deviation of 1.718 Å, confirming high structural similarity (Fig. 4a). Similar to hDHODH, *Afl*DHODH has two domains, a small N-terminal domain (L95–L128) and a large C-terminal α/β-barrel (T146-C-terminus), which are linked by an extended loop. The N-terminal domain contains two α-helices (α2, α3) with short loops. The C-terminal domain forms a canonical α/β-barrel with eight parallel β-strands (β1–β8) surrounded by nine α-helices (α4, α5, α7–α9, α11–α13, α15). Molecular docking showed that *Afl*DHODH binds orotate (ORO) and flavin mononucleotide (FMN) similarly to hDHODH, with conserved binding sites (30, 32) (Fig. 4b and c). The active sites of *Afl*DHODH are located in the core of the α/β-barrel, where it anchors dihydroorotate (DHO) via the conserved residues N210 and N379 and stabilizes FMN through N210, Y212, and Y501. During catalysis, *Afl*DHODH uses its catalytic residue S309 to abstract a proton from the C5 position of DHO and transfers a hydride from the C6 position of DHO to FMN, thereby oxidizing DHO to ORO. *Afl*DHODH also donates a proton via L349 to the N1 atom of FMN, which reduces FMN to FMNH₂. Subsequently, ubiquinone in the mitochondrial inner membrane enters the N-terminal hydrophobic channel (formed by α2 and α3), where it accepts two hydrogens from FMNH₂. In this process, ubiquinone is reduced to ubiquinol, which then detaches from the hydrophobic channel and participates in the mitochondrial electron transport chain (ETC); meanwhile, FMNH₂ is reoxidized to FMN, which returns to the FMN-binding cavity in the α/β-barrel domain to prepare for the next redox cycle (33). Notably, compared with hDHODH, *Afl*DHODH has four additional α-helices (α1, α6, α10, α14), though their functions remain unclear.

Previous studies have utilized the crystal structure of hDHODH to generate a homology model of *Af*DHODH, which shares 35% identity and 84% coverage with hDHODH across the full-length sequences (12). The binding model of *Af*DHODH with olorofim revealed that olorofim inhibits enzymatic activity by competitively occupying the ubiquinone-binding channel. Key residues mediating this interaction were predicted, including H116, V200, R202, M209, W213, and Y510. To validate these predictions, researchers constructed the mutant strain *Ca*DHODH_F162V_V171M in *Candida albicans* (a species naturally insensitive to olorofim) by replacing F162 and V171 in *Ca*DHODH with the homologous residues V200 and M209 from *Af*DHODH. Phenotypic analysis demonstrated a significant increase in olorofim susceptibility in the *Ca*DHODH_F162V_V171M mutant, confirming these residues as critical determinants of olorofim binding (12).

To understand the critical binding sites between olorofim and *Afl*DHODH, we performed molecular docking and molecular dynamics simulations based on the *Afl*DHODH structure described above. Results reveal that olorofim binds stably to *Afl*DHODH in a mode analogous to that observed in *Af*DHODH, with both localized within the N-terminal ubiquinone channel, supporting the hypothesis that olorofim may compete with ubiquinone for binding to this channel (Fig. 4d). Residues H96, V100, V101, A111, H115, L122, L125, P132, Y212, I504, and V505 form the olorofim-binding interface in *Afl*DHODH, where mutations may compromise olorofim susceptibility (Fig. 4d). Notably, residue G118 is spatially adjacent to the binding pocket. Protein-ligand interaction analysis suggests that mutating G118 to C/S/V in *Afl*DHODH may increase the side-chain bulk, potentially causing a steric clash with the acetamide carbonyl of olorofim. This hypothetical steric clash could shorten intermolecular distances from >2.5 Å to <2.3 Å, which would hinder olorofim access to the *Afl*DHODH ubiquinone channel and weaken the stability between olorofim and *Afl*DHODH (Fig. 4e).

## DISCUSSION

In this study, we confirmed that amino acid substitutions at DHODH G118 lead to olorofim resistance in *A. flavus*. Sequence alignment revealed that this residue is highly conserved across pathogenic filamentous and dimorphic fungi, suggesting that mutations at this site may confer pan-fungal olorofim resistance. *In vitro*, DHODH G118 substituted strains exhibited high-level resistance (MIC >16 µg/mL). *In vivo*, the *G. mellonella* infection model confirmed the therapeutic failure of olorofim against these mutants. Using AlphaFold-predicted DHODH models, molecular docking localized the olorofim-binding site and suggested that substitutions (G118C/S/V) may introduce steric hindrance, which could disrupt drug-enzyme interactions. These findings establish the first molecular and phenotypic profile of olorofim resistance in *A. flavus*, identifying DHODH G118 as a surveillance biomarker and urging novel strategies to counter this emerging threat.

This study provides the first comprehensive structural annotation of the functional domains, catalytic sites, and olorofim-binding pocket topology of *Afl*DHODH. Docking analysis revealed that olorofim binds at the entrance of the ubiquinone channel, blocking ubiquinone access to the redox-active site and inhibiting enzymatic activity, consistent with previous predictions for *A. fumigatus*. By defining the putative binding pocket, we identified key interacting residues corresponding to positions in *Af*DHODH homologs (e.g., H116, G119, L164, V200, M209) that are known to confer resistance (12, 22), providing strong structural support for our model. Importantly, we also revealed several *Afl*DHODH-specific interacting residues (e.g., I504, V505). Furthermore, our analysis provides unprecedented mechanistic insight into the primary resistance hotspot. While previous studies merely speculated that *Af*DHODH G119 mutations restrict channel access or cause undefined conformational changes (22), our modeling explicitly demonstrates that the *Afl*DHODH G118 substitutions introduce direct steric hindrance and thereby reduce drug-binding affinity. Additionally, we predicted that histidine 115 (H115) forms a stable π-π bond with olorofim (Fig. S4), and mutations at this site would reduce drug-enzyme binding affinity to confer resistance. However, since the AlphaFold model yields predicted structures, experimentally resolved AflDHODH crystal structures will be essential for validating these findings.

DHODH plays dual roles in pyrimidine biosynthesis and ETC activity. Localized on the inner mitochondrial membrane, it catalyzes ubiquinone reduction, which is vital for maintaining redox homeostasis and regulating reactive oxygen species levels (33). Inhibiting DHODH compromises ETC function and exacerbates oxidative stress. To assess the effect of G118 substitutions, we compared the adaptability of G118 mutants and the WT strain to cell wall-perturbing agents and oxidative stress inducers. All strains showed similar tolerance to each stressor, indicating G118 mutations do not impair adaptive responses to oxidative or cell wall stress (Fig. S3) and do not obstruct ubiquinone access to the channel. In contrast, G118 mutants displayed reduced sensitivity to olorofim, implying the mutation hinders olorofim entry into the channel. Furthermore, combining olorofim with stressors synergistically suppressed fungal growth (Fig. S3). This synergy can be explained by the known function of DHODH: by disrupting both ETC activity and pyrimidine synthesis, olorofim amplifies the metabolic damage inflicted by oxidative stress (ETC impairment) and cell wall stress (metabolic imbalance). Crucially, this finding indirectly supports our molecular docking hypothesis that olorofim and ubiquinone compete for binding to the same N-terminal channel of DHODH.

Although resistance development is often associated with reduced virulence (34), strains with G118 substitutions in our study imposed no significant fitness cost under the tested conditions and preserved fungal pathogenicity in the *G. mellonella* infection model. Consequently, proactive surveillance of G118 mutations will be critical during clinical use of olorofim to monitor treatment efficacy and guide therapeutic strategies. Notably, the cross-resistance we identified between the fungicide ipflufenoquin and the clinical antifungal olorofim carries profound public health significance, as it directly mirrors the well-documented triazole resistance crisis driven by agricultural demethylase inhibitors (DMIs). The global emergence of triazole-resistant *A. fumigatus*, driven by the widespread agricultural use of DMIs, has severely limited treatment options for immunocompromised patients with IA (5, 6). Parallel to this precedent, *in vitro* studies have shown exposure of *A. fumigatus* to ipflufenoquin selects for olorofim-resistant strains (22), and our data confirm that homologous DHODH hotspot mutations (G118 in *A. flavus*, analogous to G119 in *A. fumigatus*) mediate this cross-resistance. Therefore, the emergence of these resistant strains poses a significant public health risk, necessitating future research employing unbiased *in vitro* evolution and whole-genome sequencing to uncover novel resistance loci and compare the divergent evolutionary trajectories of *A. flavus* and *A. fumigatus* beyond the known hotspots verified in this study. To preserve the clinical efficacy of olorofim, it is imperative that agricultural and clinical sectors must strengthen collaboration to enhance supervision of ipflufenoquin use and implement proactive monitoring of resistance loci.

## SUPPLEMENTAL MATERIAL

Supplemental figures spectrum.00251-26-s0001.docx Fig. S1 to S4.

ASM does not own the copyrights to Supplemental Material that may be linked to, or accessed through, an article. The authors have granted ASM a non-exclusive, world-wide license to publish the Supplemental Material files. Please contact the corresponding author directly for reuse.
