# Supplementary material for: Amino acid substitutions at the G118 hotspot in dihydroorotate dehydrogenase confer olorofim resistance in Aspergillus flavus
Source: Microbiol Spectr. 2026 Jul 14;14(8):e00251-26. doi: 10.1128/spectrum.00251-26 (PMC13436209; doi:10.1128/spectrum.00251-26)
Supplement: Supplementary file 1 [file spectrum.00251-26-s0001.docx]

**Supplementary materials**

Figure S1. (a) Schematic representation of the CRISPR-Cas9 targeting the *Afl*DHODH locus. The 20-bp crRNA target sequence is indicated, followed immediately by the PAM sequence (GGG, highlighted in red). Single-stranded DNA (ssDNA) repair templates were designed to introduce the specific base substitutions. The GGG codon was replaced by TGT (for G118C), AGC (for G118S), or GTG (for G118V). (b) Sanger sequencing results confirming the correct point mutations introduced into the genome of the recipient strains.

Figure S2. Radial growth rates of NRRL3357, *Afl*DHODH_G118C, *Afl*DHODH_G118S, and *Afl*DHODH_G118V.

Figure S3. Inhibitory effects of olorofim (0.005 μg/mL and 4 μg/mL), Congo red (600 μg/mL), calcofluor white (300 μg/mL), SDS (0.03%), H_2_O_2_ (3 mM), and menadione (80 mM), or their combinations on the growth of *A. flavus.* Spore suspensions of *A. flavus* were serially diluted from 2 × 10^5^ to 2 spores and spot-inoculated onto the agar surface. The control group contained PDA medium alone. All plates were incubated at 35°C for 48 hours to assess growth inhibition.

Figure S4. (a) RMSD of *Afl*DHODH and olorofim through molecular dynamics simulations. (b) 2D diagram of detailed ligand-atom interactions with the protein residues.
